# Supplementary material for: A UMLS-based spell checker for natural language processing in vaccine safety
Source: BMC Med Inform Decis Mak. 2007 Feb 12;7:3. doi: 10.1186/1472-6947-7-3 (PMC1805499; doi:10.1186/1472-6947-7-3)
Supplement: Additional file 13 — RAP application directory. Sets up directory for RAP (RDF application for PHP) source code [file 1472-6947-7-3-S13.gz › rap/doc/phpdoc/__filesource/fsource_default__vocabularyDC.php.html]

xml version="1.0" encoding="iso-8859-1"?


File Source for DC.php


# Source for file DC.php

Documentation is available at DC.php

```
1. <?php
2. // ----------------------------------------------------------------------------------
3. // Dublin Core Vocabulary
4. // ----------------------------------------------------------------------------------
5. // Version                   : 0.9
6. // Authors                   : Chris Bizer (chris@bizer.de)
7. //
8. // Description               : Wrapper, defining resources for all terms of the Dublin
9. //                               Core Vocabulary. For details about DC see: http://dublincore.org/
10. //                                Using the wrapper allows you to define all aspects of
11. //                             the vocabulary in one spot, simplifing implementation and
12. //                             maintainence. Working with the vocabulary, you should use
13. //                             these resources as shortcuts in your code.
14. //
15. // ----------------------------------------------------------------------------------
16. // <BR><BR>History:<UL>
17. // <LI>07-26-2004                 : Update for latest DC recs; changed suffixed globals to
18. //                                  array items.(auer@informatik.uni-leipzig.de)</LI>
19. // <LI>02-21-2003                 : Initial version (evan@wikitravel.org)</LI>
20. // ----------------------------------------------------------------------------------


25. // DC concepts

27. $DC_contributor = new Resource(DC_NS . 'contributor');
28. $DC_coverage = new Resource(DC_NS . 'coverage');
29. $DC_creator = new Resource(DC_NS . 'creator');
30. $DC_date = new Resource(DC_NS . 'date');
31. $DC_description = new Resource(DC_NS . 'description');
32. $DC_format = new Resource(DC_NS . 'format');
33. $DC_identifier = new Resource(DC_NS . 'identifier');
34. $DC_language = new Resource(DC_NS . 'language');
35. $DC_publisher = new Resource(DC_NS . 'publisher');
36. $DC_rights = new Resource(DC_NS . 'rights');
37. $DC_source = new Resource(DC_NS . 'source');
38. $DC_subject = new Resource(DC_NS . 'subject');
39. $DC_title = new Resource(DC_NS . 'title');
40. $DC_type = new Resource(DC_NS . 'type');

42. // Dublin Core Metadata Element Set (DCMES) 1.1
43. $DCMES_name = array('contributor', 'coverage', 'creator', 'date',
44. 'description', 'format', 'identifier', 'language',
45. 'publisher', 'relation', 'rights', 'source',
46. 'subject', 'title', 'type');

48. foreach ($DCMES_name as $name) {
49. $DCMES[$name] = new Resource(DC_NS . $name);
50. $GLOBALS['DC_' . $name] = $DCMES[$name];
51. }

53. // Other Elements and Element Refinements
54. $DCTERM_name = array('abstract', 'accessRights', 'alternative', 'audience',
55. 'available', 'bibliographicCitation', 'conformsTo',
56. 'created', 'dateAccepted', 'dateCopyrighted',
57. 'dateSubmitted', 'educationLevel', 'extent',
58. 'hasFormat', 'hasPart', 'hasVersion',
59. 'isFormatOf', 'isPartOf', 'isReferencedBy', 'isReplacedBy',
60. 'isRequiredBy', 'issued', 'isVersionOf', 'license',
61. 'mediator', 'medium', 'modified', 'references',
62. 'replaces', 'requires', 'rightsHolder', 'spatial',
63. 'tableOfContents', 'temporal', 'valid');
64. foreach ($DCTERM_name as $name) {
65. $DCTERM[$name] = new Resource(DCTERM_NS . $name);
66. }

68. // Encoding schemes
69. $scheme_name = array('Box', 'DCMIType', 'IMT', 'ISO3166', 'ISO639-2',
70. 'LCC', 'LCSH', 'MESH', 'Period', 'Point',
71. 'RFC1766', 'RFC3066', 'TGN', 'UDC', 'URI',
72. 'W3CDTF');

74. foreach ($scheme_name as $name) {
75. $DCTERM[$name] = new Resource(DCTERM_NS . $name);
76. }

78. // DCMI Type Vocabulary
79. $DCMITYPE_names = array('Collection', 'Dataset', 'Event', 'Image',
80. 'InteractiveResource', 'MovingImage', 'PhysicalObject',
81. 'Service', 'Software', 'Sound', 'StillImage', 'Text');

83. foreach ($DCMITYPE_names as $name) {
84. $DCMITYPE[$name] = new Resource(DCMITYPE_NS . $name);
85. }

87. ?>
```

Documentation generated on Fri, 17 Dec 2004 16:14:41 +0100 by phpDocumentor 1.3.0RC3
